# Association between perinatal depression in mothers and the risk of childhood infections in offspring: a population-based cohort study

**DOI:** 10.1186/1471-2458-10-799

**Published:** 2010-12-31

**Authors:** Lu Ban, Jack E Gibson, Joe West, Laila J Tata

**Affiliations:** 1 Department of Epidemiology & Public Health, University of Nottingham, Nottingham, UK

## Abstract

**Background:**

Previous studies have suggested that children of mothers who experience depression during the perinatal period may have more infections, but such studies are few in number and none have been carried out in the United Kingdom (UK) population. The aim of this study was to investigate the association between perinatal depression in mothers and the risk of childhood infections in offspring in the UK general population.

**Methods:**

We used data from The Health Improvement Network (THIN), a large database of electronic primary care medical records to conduct a cohort study among all first-born singleton children born and enrolled in THIN between 1988 and 2004. We used Poisson regression to compare the incidence of gastrointestinal infections and lower respiratory tract infections reported between birth and age 4 years among children of mothers with a record of perinatal depression with those born to mothers with no such history.

**Results:**

Children of mothers with perinatal depression had a 40% increased risk of gastrointestinal infections and a 27% increased risk of lower respiratory tract infections compared with children of mothers without perinatal depression (incidence rate ratios = 1.40 and 1.27; 95% confidence intervals 1.37-1.42 and 1.22-1.32, respectively). On restricting to antibiotic-treated infections there was a slight increase in the magnitude of association with gastrointestinal infections but a decrease in that with lower respiratory tract infections (incidence rate ratios = 1.47 and 1.19; 95% confidence intervals 1.34-1.61 and 1.11-1.27, respectively).

**Conclusions:**

Maternal perinatal depression is associated with increased rates of childhood gastrointestinal infections, particularly more severe infections, and lower respiratory tract infections in the UK. Preventing maternal perinatal depression may avoid substantial morbidity among offspring, although further work is also needed to investigate the detailed reasons for these findings.

## Background

According to recent guidance from the National Institute for Health and Clinical Excellence [1], depression in mothers during and after pregnancy (maternal perinatal depression) is one of the most important issues for women's health in the United Kingdom (UK). Depression is estimated to affect 10% to 20% of women during the perinatal period in high-income countries [2-4] and can have a substantial adverse impact on women's health as well as on maternal behaviour towards their children and childcare practices [5-8]. In addition, there is some evidence to suggest that a dysregulated hapothalamic pituitary adrenocortical axis may lead to immunosupression in mothers with perinatal depression [6,9], which may directly impair their children's immune and neuroendocrine development.

Researchers in Pakistan studying 265 mothers and their children found an increased risk of diarrhoea among children born to mothers with postnatal depression compared with those born to mothers without postnatal depression (adjusted odds ratio [OR] = 3.1; 95% confidence interval [CI] 1.8-5.1) [10]. They also reported an unadjusted result for respiratory infections in children born to mothers with either antenatal or postnatal depression (OR = 1.1; 95% CI 0.9-1.4) in the same cohort [11]. Moreover, a cross-sectional study conducted in the United States of America reported an association between postpartum depression and physical illness (including, but not limited to, infections) in children (p = 0.03) [9], suggesting that an association between maternal perinatal depression and childhood infections may also exist in high-income countries.

Since the prevalence of maternal perinatal depression is high, any association with childhood infections could be an important public health concern in high-income countries. Although the relative impact of infections on childhood survival and severe morbidity is lesser than in low income countries, infectious illnesses are still a major cause of mortality and morbidity among young children in higher income countries and are both socially and economically burdensome [12,13]. In European countries in 2004, more than 16% of deaths in children under five-years of age were attributed to childhood acute respiratory infections (ARIs) and about 14% were attributed to childhood diarrhoeal illness [12].

We therefore conducted a large population-based study in the UK with the primary aim of examining the potential effect of maternal perinatal depression on childhood gastrointestinal (GI) infections and lower respiratory tract infections (LRTIs) in the first four years of life. We also investigated whether healthcare-seeking behaviour differed between mothers with and without perinatal depression, as this may lead to differential levels of diagnosis of minor self-limiting infections.

## Methods

### Dataset and study population

We used data from The Health Improvement Network (THIN), a large database of anonymised computerised primary health care records of patients throughout the UK. There is a high standard of validated recording of medical diagnoses, medical conditions and symptoms and prescriptions in THIN [14,15]. The data in this study derive from 255 general practices across England and Wales with longitudinal records of 3.9 million patients. Previous research [15,16] has shown that the population in THIN is generally representative of the UK population. Ethical approval for this study was granted by the South East Research Ethics Committee.

For each woman who was of childbearing age (15 to 50 years of age) at any time between January 1988 and November 2004, birth-related entries in their general practice records during this period were linked to registered children born in the same household at the time of delivery. Women were included if they had one or more live born children recorded in the database. For this study, we only included the first recorded singleton pregnancy ending in a live born child for each woman. Pregnancies ending in stillbirth, miscarriage, therapeutic abortion and multiple pregnancies (e.g. twins delivered) were excluded. Follow-up data were available for all children for the first four years of their life unless they died or ceased to be registered at the practice. All analyses were restricted to the first four-years of life since in the UK children generally start attending school at between 4 and 5 years of age, leading to a considerable change in their environmental exposure to infection.

We extracted data including records of GI infections, LRTIs and antibiotic prescriptions for children and records of maternal perinatal depression and antidepressant use for mothers. Based on previous research [9,10,17,18], we also extracted data (where available) on maternal age at birth, body mass index [BMI] before pregnancy, smoking status before childbirth, caesarean section delivery, and household socioeconomic status (represented by quintiles of Townsend deprivation indices [19]), and the sex of the child, since these factors may be related to maternal perinatal depression as well.

### Measurement of maternal perinatal depression

Mothers were defined as having perinatal depression if, at any time during pregnancy or in the first six months after childbirth, they had a medical record of diagnosed depression (e.g. major depression, mild depression, or chronic depression), or if they received at least one prescription for an antidepressant medication.

### Measurement of childhood infections and antibiotic prescriptions

Children were defined as having GI infections if they had at least one clinical diagnosis of GI infection (e.g. infectious colitis, enteritis or gastroenteritis) or infection-related symptoms (e.g. vomiting or diarrhoea) recorded in their general practice records. The date of recovery from illness was defined as the 15^th ^day after diagnosis. If a subsequent diagnosis was recorded prior to the recovery date, this was considered part of the same episode of illness and the recovery date was extended to the 8^th ^day after this recording (only if later than the original recovery date, ensuring that an episode lasted at least 14 days). Further recordings prior to the recovery date were treated in the same way. Recordings after the date of recovery were treated as indicating the onset of new episodes of illness. We used the same process to identify and classify episodes of LRTIs, where these were defined as at least one clinical diagnosis of an LRTI (e.g. pneumonia or bronchitis) in the primary care record. To obtain a measure of more severe episodes of illness, we also identified all antibiotic-treated GI and LRTI infectious episodes, which we defined as those where children had one or more records of a prescription for an antibiotic dated on or after the date of onset and prior to the date of recovery.

### Statistical analysis

We calculated crude incidence rates as the total number of episodes for each infectious outcome (episodes of GI infections with and without antibiotic treatment and episodes of LRTIs with and without antibiotic treatment) divided by children's total follow-up period during the first four years of life. We calculated the absolute differences in crude incidence rates by subtracting the rates in children of mothers without perinatal depression from those in children of mothers with perinatal depression. To assess whether the incidence rates changed over the first years of the child's life and whether the association of maternal perinatal depression with childhood infections changed with increasing time from birth, we also calculated the incidence rates for each year of the child's life separately.

To estimate the effect of maternal perinatal depression on the risk of childhood infections, we used Poisson regression to obtain incidence rate ratios (IRRs) for each type of infectious outcome among children of mothers with perinatal depression relative to children of mothers without perinatal depression, overall and for each year of age up to 4 years. Findings were considered statistically significant where p was less than 0.05.

We also attempted to assess whether mothers with perinatal depression might be more likely to report childhood illness to their general practitioners (GPs), or to do so sooner than mothers without perinatal depression, by examining whether time to first polio immunisation in children differed between mothers with and without perinatal depression as a potential marker of mothers' healthcare-seeking behaviour. We determined the date of each child's first polio immunisation as recorded in the general practice records for children born between 1^st ^January 1991 and 31^st ^December 2003 only, since the UK immunisation schedule and vaccine type did not change during this time period [20,21]. We calculated a median time to first polio vaccination in children of mothers with and without perinatal depression. All analyses were carried out using Stata SE 10.0 software.

### Statistical Power

Based on previous studies [2-4,22], we estimated that about 10% of mothers in our study population would have perinatal depression. We calculated that our sample size would give us over 99% power to detect a 1.5-fold increased rate in children of mothers with perinatal depression for both GI infections and LRTIs.

## Results

### Study population

We identified a cohort of 107,587 mothers and their first live born children. Of the mothers, 9,722 (9.1%) had perinatal depression. The mean age of the mothers was 29 years (Standard Deviation 5.7). Complete follow-up data to age 4 years was available for 80.5% of all children, and 96.0% of children were followed up to at least 2 years of age.

Table 1 shows that compared with mothers without perinatal depression, mothers with perinatal depression were slightly younger (p < 0.001). They were also more likely to be current smokers, obese, in a household with higher material deprivation and to have had a caesarean section delivery than mothers without perinatal depression (p < 0.001 for all associations).

**Table 1:** Characteristics of mothers with and without depression (N = 107587)

	Maternal perinatal depression*			
	
	No (n = 97,815) N (%)	Yes (n = 9,772) N (%)	χ^2^(df^†^)	P
Maternal age at birth of child (years)				
Mean (Standard deviation)	29.2 (5.6)	28.6 (5.9)	t^α ^= 9.5 (107585)	< 0.001
Maternal body mass index (kg/m^2^)				
Normal (18.5-24.9)	37971 (38.8)	4069 (41.6)	575.9 (4)	< 0.001
Underweight (<18.5)	2660 (2.7)	395 (4.0)		
Overweight (25-29.9)	12093 (12.4)	1502 (15.4)		
Obese (>30)	5653 (5.8)	910 (9.3)		
Missing	39438 (40.3)	2896 (29.6)		
Maternal smoking				
Non-smoker	47266 (48.3)	3952 (40.4)	1300 (3)	< 0.001
Ex-smoker	4698 (4.8)	567 (5.8)		
Current smoker	19985 (20.4)	3463 (35.4)		
Missing	25866 (26.4)	1790 (18.3)		
Household socioeconomic status				
1 (Least deprivation)	16028 (16.4)	1112 (11.4)	419.1 (5)	< 0.001
2	11714 (12.0)	972 (10.0)		
3	11170 (11.4)	1106 (11.3)		
4	9298 (9.5)	1214 (12.4)		
5 (Most deprivation)	7063 (7.2)	1078 (11.0)		
Missing	42542 (43.5)	4290 (43.9)		
Caesarean section delivery	13604 (13.9)	1616 (16.5)	50.6 (1)	< 0.001
Sex of the child				
Male	50192 (51.3)	4922 (50.4)	3.2 (1)	0.075

* Mothers with either depression diagnosis and/or at least one antidepressant prescription either during pregnancy and/or in the first six months after the child's delivery.

^†^Degrees of freedom

^α ^Student t-test statistic

### Maternal depression and childhood infections

Proportionately more children born to mothers with perinatal depression had at least one GI infection (60.6%) than children born to mothers without perinatal depression (49.2%). Similarly, children born to mothers with perinatal depression were more likely to have one or more LRTIs than children born to mothers without perinatal depression (20.9% vs. 17.0%). When only episodes of childhood infections with at least one antibiotic prescription were considered, the prevalence decreased to 5.2% and 3.6% for GI infections and 10.2% and 8.7% for LRTIs, respectively.

The overall rates of childhood GI infections were 3.41 per 10 person-years (95% CI 3.35-3.47) for children of mothers with perinatal depression and 2.44 per 10 person-years (95% CI 2.43-2.46) for children of mothers without perinatal depression (Table 2). The overall rates for childhood LRTIs were 0.91 per 10 person-years (95% CI 0.88-0.94) and 0.72 per 10 person-years (95% CI 0.71-0.73), respectively for mothers with and without perinatal depression (Table 2). The overall rate differences were therefore 0.97 per 10 person-years (95% CI 0.90-1.03) for GI infections and 0.19 per 10 person-years (95% CI 0.16-0.23) for LRTIs (Table 2).

**Table 2:** Associations between maternal perinatal depression and childhood gastrointestinal infections and childhood lower respiratory infections

	Maternal perinatal depression					
	
	No		Yes			
			
Childhood infections	Events	Rate per 10 person-years (95% CI*)	Events	Rate per 10 person-years (95% CI*)	Rate difference (95% CI*)	Unadjusted IRR^# ^ (95% CI*)
GI infections^†^						
Age (years)						
0-4	84999	2.44 (2.43-2.46)	11634	3.41 (3.35-3.47)	0.97 (0.90-1.03)	1.40 (1.37-1.42)
*0-1*	34928	3.71 (3.67-3.75)	4950	5.31 (5.16-5.46)	1.60 (1.45-1.75)	1.43 (1.39-1.48)
*1-2*	26845	3.02 (2.98-3.06)	3533	4.06 (3.93-4.19)	1.04 (0.90-1.18)	1.34 (1.30-1.39)
*2-3*	14289	1.69 (1.66-1.72)	1981	2.40 (2.30-2.51)	0.71 (0.60-0.82)	1.42 (1.35-1.49)
*3-4*	8937	1.11 (1.09-1.13)	1170	1.49 (1.41-1.58)	0.38 (0.30-0.47)	1.35 (1.27-1.43)
LRTIs^‡^						
Age (years)						
0-4	25143	0.72 (0.71-0.73)	3139	0.91 (0.88-0.94)	0.19 (0.16-0.23)	1.27 (1.22-1.32)
*0-1*	12202	1.28 (1.26-1.31)	1632	1.72 (1.64-1.81)	0.44 (0.36-0.53)	1.35 (1.28-1.42)
*1-2*	6530	0.73 (0.71-0.75)	725	0.82 (0.76-0.88)	0.09 (0.03-0.16)	1.13 (1.05-1.22)
*2-3*	3783	0.45 (0.43-0.46)	454	0.55 (0.50-0.60)	0.10 (0.05-0.15)	1.23 (1.11-1.35)
*3-4*	2628	0.33 (0.31-0.34)	328	0.42 (0.37-0.46)	0.09 (0.04-0.14)	1.28 (1.14-1.44)

* Confidence interval

^# ^Incidence rate ratio

^† ^Gastrointestinal infections

^‡ ^Lower respiratory tract infections

In addition, the rates of childhood infections in children of mothers with perinatal depression were consistently higher than those of mothers without perinatal depression through the first four years of life, generally decreasing gradually in both cohorts for both GI infections and LRTIs as the children got older (Table 2).

In our study, children born to women with perinatal depression were 40% more likely to have GI infections and 27% more likely to have LRTIs compared with those born to women without perinatal depression (IRR = 1.40; 95% CI 1.37-1.42 and IRR = 1.27; 95% CI 1.22-1.32, respectively). The observed effects were similar for each year within the first four years of the child's life (Table 2). Furthermore, for more severe infectious episodes that were treated with antibiotics (Table 3), the effect of maternal perinatal depression on childhood infections increased slightly for GI infections and decreased slightly for LRTIs (IRR = 1.47; 95% CI 1.34-1.61 and IRR = 1.19; 95% CI 1.11-1.27, respectively).

**Table 3:** Associations between maternal perinatal depression and childhood infections with antibiotic treatment

	Maternal perinatal depression					
	
	No		Yes			
			
Antibiotic-treated childhood infections	Events	Rate per 10 person-years (95% CI*)	Events	Rate per 10 person-years (95% CI*)	Rate difference (95% CI*)	Unadjusted IRR^# ^ (95% CI*)
GI infections^†^						
Age (years)						
0-4	3550	0.10 (0.10-0.10)	512	0.15 (0.14-0.16)	0.05 (0.03-0.06)	1.47 (1.34-1.61)
*0-1*	1144	0.12 (0.11-0.13)	176	0.18 (0.16-0.21)	0.07 (0.04-0.09)	1.54 (1.32-1.81)
*1-2*	1153	0.13 (0.12-0.14)	172	0.19 (0.17-0.23)	0.07 (0.04-0.10)	1.52 (1.29-1.78)
*2-3*	731	0.09 (0.08-0.09)	104	0.12 (0.10-0.15)	0.04 (0.01-0.06)	1.45 (1.18-1.78)
*3-4*	522	0.06 (0.06-0.07)	60	0.08 (0.06-0.10)	0.01 (0.00-0.03)	1.18 (0.90-1.54)
LRTIs^‡^						
Age (years)						
0-4	8868	0.25 (0.25-0.26)	1035	0.30 (0.28-0.32)	0.05 (0.03-0.07)	1.19 (1.11-1.27)
*0-1*	3462	0.36 (0.35-0.37)	418	0.44 (0.40-0.48)	0.07 (0.03-0.12)	1.21 (1.10-1.34)
*1-2*	2536	0.28 (0.27-0.29)	276	0.31 (0.28-0.35)	0.03 (0.00-0.07)	1.11 (0.98-1.25)
*2-3*	1661	0.20 (0.19-0.21)	195	0.23 (0.20-0.27)	0.04 (0.00-0.07)	1.20 (1.03-1.39)
*3-4*	1209	0.15 (0.14-0.16)	146	0.19 (0.16-0.22)	0.04 (0.00-0.07)	1.24 (1.04-1.47)

* Confidence interval

^# ^Incidence rate ratio

^† ^Gastrointestinal infections

^‡^Lower respiratory tract infections

### Maternal perinatal depression and the time to first polio vaccination in children

We identified 92,218 children born between 1^st ^January 1991 and 31^st ^December 2003 who accounted for 85.7% of the whole cohort and who were representative of the whole cohort as they had almost identical distributions of maternal characteristics (data not shown). Overall, 98% of children born to women with perinatal depression had their first polio vaccination, as did the same proportion of children born to women without perinatal depression. The median time of receipt of the first polio vaccination was 61 days after birth for children of mothers with perinatal depression (interquartile range [IQR] 58-68) and this was almost identical for children of mothers without perinatal depression (median = 61 days; IQR 57-67).

## Discussion

### Principal findings

Our results show that children born to women with perinatal depression had a 40% increased rate of GI infections and a 27% increased rate of LRTIs compared with children born to women without perinatal depression. For more severe, antibiotic-treated infections, the observed effects increased slightly for GI infections to 47% but decreased slightly for LRTIs to 19%. Our observed absolute differences in rates suggest that over a one year period there were about 97 more GI infections and 19 more LRTIs among every 1000 children born to women with perinatal depression than among 1000 children born to women without perinatal depression. Moreover, the near identical timing of the first polio vaccination among both groups provides no evidence to support the possibility that higher rates of GI infections and LRTIs in children of mothers with perinatal depression may be explained by differential levels of diagnosis due to increased healthcare-seeking behavior among this group.

### Strengths and limitations

We have conducted a large population-based cohort study of the association between maternal perinatal depression and childhood infections using primary care data in the UK. Our considerable sample size means that our findings are unlikely to be due to chance. The data we used were obtained from a national database and prospectively recorded by GPs, thus excluding the possibility of recording or recall bias in both our exposure and outcome.

Full 4-year follow-up data were not available for 19.5% of children. Such drop-outs occur due to death or patients deregistering from GP practices. There was no evidence of an increased risk of death in either cohort. In the UK registration with a general practitioner is continuous unless a patient actively requests removal from the practice list, or makes an application to join another practice (typically due to a change of address, or the opening of a new practice). Drop-out rates were similar in both cohorts and we feel they are unlikely to have had a substantial impact on our results.

Although the inclusion of postpartum depression in the exposure definition meant that there could be potential for reverse causation due to some overlap between postpartum depression and childhood infections in the first six months of a child's life, such overlap could not affect the observations at age 1, 2, and 3 years. As we found that the increased risk of childhood infections in mothers with perinatal depression was consistent throughout the first four years of the child's life, we believe it is unlikely that our results were due to reverse causality.

There was a legitimate weakness in our exposure measurement as a certain proportion of mothers with perinatal depression are neither diagnosed nor treated in primary care. The prevalence of diagnosed maternal perinatal depression found in our study (about 9.1%) was in general 4-6% lower than some previous studies conducted in high-income countries [2-4,23]. However, these studies were based in research settings where selected populations were screened for depression mostly using self-administered questionnaires and their samples were much smaller than ours. On restricting to diagnosed depression using a standardized interviewing schedule, two previous studies (including a meta-analysis) [4,22] found fairly similar prevalence figures to ours. Although we acknowledge that existing depression does remain undiagnosed in the population, during pregnancy and in the first few months after delivery, women typically have more frequent contact with health professionals than they would usually do because of antenatal/postnatal check-ups. This would tend to reduce under-diagnosis of depression this period. Nevertheless, the presence of under-diagnosis in this population would only result in an underestimation of the risk of childhood GI infections and LRTIs in children associated with perinatal depression, since such misclassification would produce a bias towards the null-hypothesis (of no association between perinatal depression in mothers and childhood infections in offspring).

We defined childhood infections as children having been diagnosed with GI infections or LRTIs in primary care, which primarily relied on mothers bringing their children to see their GPs, so they most likely represented the more severe end of the spectrum of these infections. We supposed that, if women with perinatal depression felt more anxious or less capable to take care of their children, they may take children to see their GPs more often than women without perinatal depression and the increased risk of childhood infections in mothers with perinatal depression therefore could be an overestimate due to biased ascertainment. The first childhood polio vaccination is a routine appointment common to both cohorts of children and mothers and one might expect the timing to be earliest and uptake the greatest amongst children of mothers with high levels of healthcare-seeking behaviour. Although timing and uptake of polio vaccination were all but identical in both cohorts we accept that this is only an indirect, measure of mothers' health seeking behaviour and we cannot conclusively exclude the possibility of biased ascertainment.

Although we considered other factors related to maternal perinatal depression in our analysis, the records of a substantial number of individuals did not provide information about factors such as household socioeconomic status (44% missing), maternal smoking (26.4%) and maternal BMI (40% missing). We were also unable to adjust for some other factors, such as the period of breastfeeding and the child's birth weight which have previously been linked to childhood infections [13,24,25] in low-income countries, because birth weight and breastfeeding status are seldom recorded in THIN. It could be argued, however, that these factors may form part of a causal pathway linking maternal perinatal depression to childhood infections. After adjusting for these factors, one previous study [10] (carried out in Pakistan) still found a statistically significant association between maternal postpartum depression and childhood GI infections, suggesting that incomplete adjustment for confounding is unlikely to completely explain the observations of our study.

### Interpretation in context of previous studies

Although to our knowledge there are only three previous studies [9-11] examining the potential association between maternal perinatal depression and childhood infections, all have found an increased risk of childhood infections in children born to women with perinatal depression. For example, in a recent American cross-sectional study of 194 mothers and their children at 4-6 weeks postpartum, Groer and Morgan found that children of mothers with postpartum depression experienced more physical illness (including diarrhoeal illness) since birth compared with children of mothers without postpartum depression (p = 0.03) [9]. This study, however, did not provide any measure of effect and did not adjust for potential confounding factors.

In our study, we combined the period of antenatal and postnatal depression together and therefore we did not examine whether the increased risk of childhood infections was independently associated with antenatal depression or early postnatal depression. Rahman *et al *carried out a similar cohort study [11] of 265 mothers (130 mothers with perinatal depression) in Pakistan and found an increased risk of diarrhoea and a slightly increased, but not statistically significant, risk of ARIs in children of mothers with antenatal or postnatal depression compared with children of mothers without depression (OR = 2.4; 95% CI 1.7-3.3 and OR = 1.1; 95% CI 0.9-1.4, respectively), although they did not adjust for any confounding factors. A subsequent study by the same authors [10] which focused only on the relationship between postnatal depression and childhood diarrhoeal illness showed a similar result to the first, although the risk of diarrhoea in children of mothers with postnatal depression compared with children of mothers without postnatal depression was much stronger once adjusted for important maternal and child factors (unadjusted OR = 2.3; 95% CI 1.6-3.1 and adjusted OR = 3.1; 95% CI 1.8-5.6). The latter study used data from the same cohort of women and only about 5% of women with antenatal depression did not also have postnatal depression [10].

Compared with our study, Rahman *et al *found a larger effect on GI infections but a slightly smaller one on respiratory infections. Their study population was from a rural area of Pakistan where childhood infections were much more common than in our study population. Furthermore, they used only binary outcomes of more or fewer than five childhood diarrhoeal episodes and more or fewer than six ARI episodes per year, while our outcomes were incidence rates of individual episodes of GI infections and LRTIs. Consequently, our results are not directly comparable. In spite of different study populations and different measures of outcome, we both found an increased risk of GI infections (and also a similar magnitude of respiratory infection risk) in children born to women with perinatal depression compared with those born to women without perinatal depression.

There could be several potential explanations for the association between perinatal depression in mothers and childhood infections in offspring. First of all, previous research [6,9] has found that mothers with depression during the perinatal period have a dysregulated hypothalamic pituitary adrenocotical axis resulting in decreased levels of salivary cortisol and potentially depressed cellular immunity. This may have a direct impact on the child's immune and neuroendocrine development. Secondly, perinatal depression can have a considerable impact on mothers' childcare abilities. Previous studies [9,26] reported that mothers with postpartum depression were less likely to breastfeed their children and more likely to give up exclusive breastfeeding than mothers without depression. Breastfeeding can strengthen the immune system of the child and it has been shown that children who are not breastfed have more acute respiratory infections than those who are not [13]. Likewise, children of mothers with perinatal depression have been found to be less well nourished than those of mothers without depression [27,28], which will increase susceptibility to infection.

In addition, postpartum depression in mothers can lead to child neglect and poorer childcare practices, especially in high-income countries [5,29,30]. Children may thus be more exposed to unsanitary and dangerous environments, increasing the potential for exposure to sources of infection.

## Conclusions

Our findings show that depression in mothers during and after pregnancy is associated with an increased risk of childhood infections in offspring. If causally related, our findings suggest that a substantial social and economic burden of childhood morbidity could be avoided through effective prevention of antenatal and postnatal depression [1], although further work is required to investigate the detailed reasons for these findings. In primary care, physicians and other healthcare professionals should be aware of the potential increased presentation of childhood infections in children of mothers with perinatal depression.

## Abbreviations

95% CI: 95% confidence interval; ARIs: acute respiratory infections; BMI: body mass index; GI infections: gastrointestinal infections; GP: general practitioner; HR: hazard ratio; IQR: interquartile range; IRR: incidence rate ratio; LRTIs: lower respiratory tract infections; OR: odds ratio; THIN: The Health Improvement Network; UK: United Kingdom.

## Competing interests

The authors declare that they have no competing interests.

## Authors' contributions

LB carried out the literature review and participated in the development of the study design, data management, data analysis, interpretation of the data, writing the article and revising article drafts for publication. JEG had the initial idea for the study and its design and participated in the development of the study design, data management, interpretation of the data, and revising article drafts for publication. JW participated in the development of the study design, interpretation of the data, and revising article drafts for publication. LJT had the initial idea for the study and its design and participated in the development of the study design, data management, interpretation of the data, and revising article drafts for publication. All authors read and approved the final manuscript.

## Pre-publication history

The pre-publication history for this paper can be accessed here:

http://www.biomedcentral.com/1471-2458/10/799/prepub

## References

[B1] National Institute for Health and Clinical Excellence Antenatal and postnatal mental health (Clinical management and service guidance) 2007 London 31990493

[B2] Gavin NI Gaynes BN Lohr KN Meltzer-Brody S Gartlehner G Swinson T Perinatal depression: a systematic review of prevalence and incidence Obstet Gynecol 2005 106 5 Pt 1 1071 1083 10.1097/01.AOG.0000183597.31630.db 16260528

[B3] Cooper PJ Murray L Hooper R West A The development and validation of a predictive index for postpartum depression Psychol Med 1996 26 3 627 634 10.1017/S0033291700035698 8733220

[B4] O'hara MW Swain AM Rates and risk of postpartum depression--a meta-analysis International Review of Psychiatry 1996 8 1 37 54

[B5] McLennan JD Kotelchuck M Parental prevention practices for young children in the context of maternal depression Pediatrics 2000 105 5 1090 1095 10.1542/peds.105.5.1090 10790467

[B6] Groër M Davis M Casey K Short B Smith K Groër S Neuroendocrine and immune relationships in postpartum fatigue MCN Am J Matern Child Nurs 2005 30 2 133 138 15775810 10.1097/00005721-200503000-00012

[B7] Turner C Boyle F O'Rourke P Mothers' health post-partum and their patterns of seeking vaccination for their infants Int J Nurs Pract 2003 9 2 120 126 10.1046/j.1322-7114.2003.00410.x 12694481

[B8] Bartlett SJ Krishnan JA Riekert KA Butz AM Malveaux FJ Rand CS Maternal depressive symptoms and adherence to therapy in inner-city children with asthma Pediatrics 2004 113 2 229 237 10.1542/peds.113.2.229 14754931

[B9] Groer MW Morgan K Immune, health and endocrine characteristics of depressed postpartum mothers Psychoneuroendocrinology 2007 32 2 133 139 10.1016/j.psyneuen.2006.11.007 17207585

[B10] Rahman A Bunn J Lovel H Creed F Maternal depression increases infant risk of diarrhoeal illness: --a cohort study Arch Dis Child 2007 92 1 24 28 10.1136/adc.2005.086579 16966339 PMC2083137

[B11] Rahman A Iqbal Z Bunn J Lovel H Harrington R Impact of maternal depression on infant nutritional status and illness: a cohort study Arch Gen Psychiatry 2004 61 9 946 952 10.1001/archpsyc.61.9.946 15351773

[B12] World Health Organisation The global burden of disease: 2004 update 2008 Geneva

[B13] Graham NM The epidemiology of acute respiratory infections in children and adults: a global perspective Epidemiol Rev 1990 12 149 178 2286216 10.1093/oxfordjournals.epirev.a036050

[B14] Lewis JD Schinnar R Bilker WB Wang X Strom BL Validation studies of the health improvement network (THIN) database for pharmacoepidemiology research Pharmacoepidemiol Drug Saf 2007 16 4 393 401 10.1002/pds.1335 17066486

[B15] Bourke A Dattani H Robinson M Feasibility study and methodology to create a quality-evaluated database of primary care data Inform Prim Care 2004 12 3 171 177 15606990 10.14236/jhi.v12i3.124

[B16] Tata L Hubbard R McKeever T Smith C Doyle P Smeeth L West J Lewis SA Fertility Rates in Women with Asthma, Eczema, and Hay Fever: A General Population-based Cohort Study Am J Epidemiol 2007 165 9 1023 1030 10.1093/aje/kwk092 17255115

[B17] Rahman A Lovel H Bunn J Iqbal Z Harrington R Mothers' mental health and infant growth: a case-control study from Rawalpindi, Pakistan Child Care Health Dev 2004 30 1 21 27 10.1111/j.1365-2214.2004.00382.x 14678308

[B18] Rahman A Harrington R Bunn J Can maternal depression increase infant risk of illness and growth impairment in developing countries? Child Care Health Dev 2002 28 1 51 56 10.1046/j.1365-2214.2002.00239.x 11856187

[B19] Morris R Carstairs V Which deprivation? A comparison of selected deprivation indexes J Public Health Med 1991 13 4 318 326 1764290

[B20] Dobson R UK health officials launch "five in one" vaccine for babies BMJ 2004 329 7462 365 10.1136/bmj.329.7462.365 15310591 PMC509334

[B21] Department of Health Poliomyelitis Immunisation Against Infectious Disease -'The Green Book' 2006 edition 2007 Norwich: The Stationery Office 313 328

[B22] Cox JL Murray D Chapman G A controlled study of the onset, duration and prevalence of postnatal depression Br J Psychiatry 1993 163 27 31 10.1192/bjp.163.1.27 8353695

[B23] Kumar R Robson KM A prospective study of emotional disorders in childbearing women Br J Psychiatry 1984 144 35 47 10.1192/bjp.144.1.35 6692075

[B24] Pelletier DL Frongillo EA Schroeder DG Habicht JP The effects of malnutrition on child mortality in developing countries Bull World Health Organ 1995 73 4 443 448 7554015 PMC2486780

[B25] Tupasi TE Mangubat NV Sunico ME Magdangal DM Navarro EE Leonor ZA Lupisan S Medalla F Lucero MG Malnutrition and acute respiratory tract infections in Filipino children Rev Infect Dis 1990 12 Suppl 8 S1047 1054 2270404 10.1093/clinids/12.supplement_8.s1047

[B26] Hasselmann MH Werneck GL Silva CVCD Symptoms of postpartum depression and early interruption of exclusive breastfeeding in the first two months of life Cad Saude Publica 2008 24 Suppl 2 S341 352 18670714 10.1590/s0102-311x2008001400019

[B27] de Miranda CT Turecki G Mari JDJ Andreoli SB Marcolim MA Goihman S Puccini R Strom BL Berlin JA Mental health of the mothers of malnourished children Int J Epidemiol 1996 25 1 128 133 10.1093/ije/25.1.128 8666480

[B28] Stewart RC Maternal depression and infant growth: a review of recent evidence Matern Child Nutr 2007 3 2 94 107 10.1111/j.1740-8709.2007.00088.x 17355442 PMC6860855

[B29] Beck CT The effects of postpartum depression on maternal-infant interaction: a meta-analysis Nurs Res 1995 44 5 298 304 10.1097/00006199-199509000-00007 7567486

[B30] Windham AM Rosenberg L Fuddy L McFarlane E Sia C Duggan AK Risk of mother-reported child abuse in the first 3 years of life Child Abuse & Neglect 2004 28 6 647 669 10.1016/j.chiabu.2004.01.003 15193853

